# Use of Home Telemonitoring to Support Multidisciplinary Care of Heart Failure Patients in Finland: Randomized Controlled Trial

**DOI:** 10.2196/jmir.3651

**Published:** 2014-12-11

**Authors:** Anna-Leena Vuorinen, Juha Leppänen, Hannu Kaijanranta, Minna Kulju, Tiina Heliö, Mark van Gils, Jaakko Lähteenmäki

**Affiliations:** ^1^VTT Technical Research Centre of FinlandTampereFinland; ^2^VTT Technical Research Centre of FinlandEspooFinland; ^3^Helsinki University Central HospitalHeart and Lung CenterHelsinkiFinland

**Keywords:** heart failure, telemonitoring, hospitalization, user experience, clinical outcomes, EHFSBS, health care resources

## Abstract

**Background:**

Heart failure (HF) patients suffer from frequent and repeated hospitalizations, causing a substantial economic burden on society. Hospitalizations can be reduced considerably by better compliance with self-care. Home telemonitoring has the potential to boost patients’ compliance with self-care, although the results are still contradictory.

**Objective:**

A randomized controlled trial was conducted in order to study whether the multidisciplinary care of heart failure patients promoted with telemonitoring leads to decreased HF-related hospitalization.

**Methods:**

HF patients were eligible whose left ventricular ejection fraction was lower than 35%, NYHA functional class ≥2, and who needed regular follow-up. Patients in the telemonitoring group (n=47) measured their body weight, blood pressure, and pulse and answered symptom-related questions on a weekly basis, reporting their values to the heart failure nurse using a mobile phone app. The heart failure nurse followed the status of patients weekly and if necessary contacted the patient. The primary outcome was the number of HF-related hospital days. Control patients (n=47) received multidisciplinary treatment according to standard practices. Patients’ clinical status, use of health care resources, adherence, and user experience from the patients’ and the health care professionals’ perspective were studied.

**Results:**

Adherence, calculated as a proportion of weekly submitted self-measurements, was close to 90%. No difference was found in the number of HF-related hospital days (incidence rate ratio [IRR]=0.812, *P*=.351), which was the primary outcome. The intervention group used more health care resources: they paid an increased number of visits to the nurse (IRR=1.73, *P*<.001), spent more time at the nurse reception (mean difference of 48.7 minutes, *P*<.001), and there was a greater number of telephone contacts between the nurse and intervention patients (IRR=3.82, *P*<.001 for nurse-induced contacts and IRR=1.63, *P*=.049 for patient-induced contacts). There were no statistically significant differences in patients’ clinical health status or in their self-care behavior. The technology received excellent feedback from the patient and professional side with a high adherence rate throughout the study.

**Conclusions:**

Home telemonitoring did not reduce the number of patients’ HF-related hospital days and did not improve the patients’ clinical condition. Patients in the telemonitoring group contacted the Cardiology Outpatient Clinic more frequently, and on this way increased the use of health care resources.

**Trial Registration:**

Clinicaltrials.gov NCT01759368; http://clinicaltrials.gov/show/NCT01759368 (Archived by WebCite at http://www.webcitation.org/6UFxiCk8Z).

##  Introduction

Heart failure (HF) is a serious and costly disease associated with poor quality of life [1], a wide range of comorbidities [2], and a high rate of hospitalization [3]. Nearly 25% of patients are readmitted within 30 days [4], and by 6 months, the proportion increases to 50% [5]. Hospitalizations cause a heavy economic burden since they are responsible for 60-70% of the total costs of HF care [6]. Moreover, the 1-year mortality of HF patients is 30% [3], and the 5-year survival rate is poorer than in most cancers [7].

A multidisciplinary care approach to heart failure is incorporated with European and American guidelines. The multidisciplinary care model includes specially trained HF nurses, the education of patients (and caregivers) regarding precipitating factors and the need for compliance with medication and diet, follow-up monitoring by trained staff, and access to specialized HF clinics [8]. Non-compliance with medication and other lifestyle recommendations is a major problem among HF patients resulting in worsening symptoms that can lead to readmission [9]. Hospitalizations may be preventable by up to 50% mainly by improving compliance with self-care [10].

Care-delivery models that incorporate telemonitoring as a part of HF patients’ care have the potential to boost patients’ compliance with self-care while at the same time bringing health care services closer to them. Meta-analyses from the years 2009-2011 link telemonitoring with improved survival, decreased hospitalizations, and improved quality of life [11-13]. However, since these meta-analyses were carried out there have been two large randomized controlled trials that have failed to show evidence in favor of telemonitoring in terms of reducing hospitalizations and death [14,15]. Similar findings have been reported in earlier studies [16,17] and more recently in smaller studies [18-20], except in the TEN-HMS trial [16], in which mortality was found to be lower in the telemonitoring group compared to usual care. Furthermore, results from the recent Whole Systems Demonstrator (WSD) study, a multisite trial involving 3230 chronically ill patients shows contradictive evidence. Telehealth was found to reduce mortality and emergency admission rates in the secondary care [21] but failed to improve quality of life or psychological outcomes [22], nor was it cost-effective [23]. Among patients with social care needs in WSD study, telecare did not alter the use of health and social care service or mortality [24]. To summarize, the literature shows conflicting evidence on the effectiveness of telemonitoring dependent on the target population and study environment and the implementation and structure of the intervention itself.

The current literature does not cover the evaluation of telemonitoring as a part of multidisciplinary care. The objective of this study was to investigate whether the multidisciplinary care of HF patients could be improved with telemonitoring at the Cardiology Outpatient Clinic of Helsinki University Central Hospital (HUCH), primarily in terms of reducing HF-related hospitalizations. We hypothesized that telemonitoring improves patients’ adherence to self-care—something that will be realized as decreased hospitalizations.

##  Methods

###  Study Design

Heart at Home was a two-arm randomized controlled trial conducted at the Cardiology Outpatient Clinic of HUCH in 2010-2012 (NCT01759368). The study protocol was approved by the Ethics Committee of the Hospital District of Helsinki and Uusimaa. All the patients provided a written informed consent before they were randomized. (See Multimedia Appendix 1 for the CONSORT EHEALTH checklist [25]).

Matched pair design was used in the randomization. The eligible patients, who were similar in left ventricular ejection fraction, NYHA classification, age, and gender, respectively, were matched in pairs. One was randomized to the control group and the other to the intervention group.

The study was divided into two parts. The first 30 intervention patients and 29 control patients started stepwise from November 2010 to February 2011. After the first 59 patients had finished their follow-up, the second group (17 intervention patients and 18 control patients) started in May to August 2011. The nominal follow-up time was 6 months. The study was completed in February 2012.

### Participants

Patients suffering from chronic heart failure were recruited to the study. The inclusion criteria were (1) diagnosis of systolic heart failure, (2) age of 18-90 years, (3) NYHA class ≥2 (an interview-based classification by the New York Heart Association concerning limitations to physical activity), (4) left ventricular ejection fraction ≤35% as measured during hospital visits, (5) need for a regular check-up visit, and (6) time from the last visit of less than 6 months. Patients were not eligible if they had a planned major medical operation, had severe comorbidity such as cancer, had participated in another clinical trial during the last 3 months, or were suspected of poor compliance. The assessment of compliance was based on patient’s technical skills, such as ability to use a mobile phone.

The electronic patient database of HUCH was used for the initial screening of patients with chronic heart failure so as to further assess their eligibility. Eligible patients were informed about the study and were asked whether they were willing to participate (and their formal consent was obtained) when they came for their normal follow-up visit. For willing patients, anthropometric and laboratory measurements were taken and the patients completed the study questionnaires. For each patient, the medication was checked and optimized. The same procedure was repeated at the end-point visit.

### Usual Care

At the Cardiology Outpatient Clinic of HUCH, there are about 600 HF patients, of whom 150-200 patients have serious heart failure that requires regular follow-up visits. A multidisciplinary care approach including patient guidance and support for self-care has been adopted at the clinic. In the care of these HF patients, the cardiac team plays a central role in monitoring and interpreting patient symptoms, optimizing medication, and providing education. The cardiac team consists of 2 physicians, a specialized heart failure nurse, and a physiotherapist who helps after a hospitalization period. As part of the care process, patients capable of carrying out self-care were identified and encouraged to regularly measure their blood pressure, heart rate, and weight at home. The information exchange between HF patients and care personnel took place during patients’ visits to the clinic and by telephone. Systematic collection and exploitation of the self-measurement data was difficult since it depended on the patient’s own activity. Often a patient had not monitored their health parameters as agreed or had forgotten to bring along the measurement notes. The heart failure nurse contacted patients by telephone if agreed in the care plan to motivate and remind them to comply with the self-care plan.

### Intervention: A Telemonitoring-Assisted Self-Care Model

For patients in the intervention arm, a new care process was introduced in which a patient regularly reported their most important health parameters to the nurse using a mobile phone app. At the beginning of the study, the patients were given a home-care package including a weight scale, a blood pressure meter, a mobile phone, and self-care instructions. The patients were advised to carry out and report the measurements together with the assessment of symptoms once a week.

**Figure 1:**
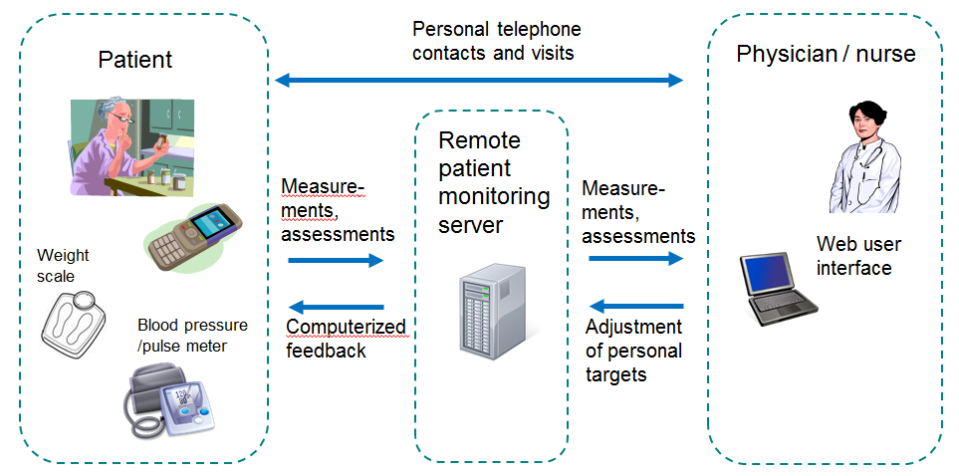
Overall architecture for remote patient monitoring.

**Figure 2:**
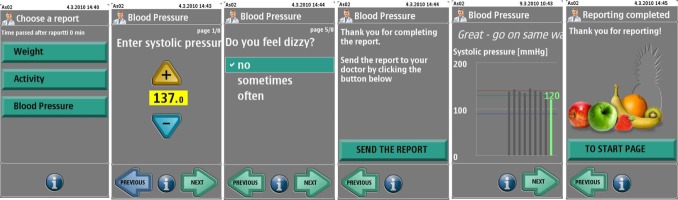
Screenshots of the reporting process with the mobile app.

A pre-installed software app on the mobile phone supported the uploading of measurements and the self-assessment of symptoms. In the development of the mobile app, particular care was paid to the simplicity of the user interface and its ease of use, since most of the patients were elderly. The measurements taken at home to be uploaded were diastolic and systolic blood pressure, pulse, body weight, and an assessment of symptoms. The symptom assessment concerned the patient’s feelings of dizziness, dyspnea, palpitation, weakness, and edema. Patients were also asked to evaluate their overall condition, that is, whether their condition had deteriorated, improved, or remained unchanged. In the context of each submission of information, the patient received automatic machine-based feedback of whether the reported parameter was within their personal targets set by the nurse. The overall architecture used in the self-care process and screenshots of the software app are depicted in Figures 1 and 2. The system was developed by VTT Technical Research Centre of Finland.

The measurements were stored on the secured remote patient monitoring server. The cardiac team was able to access the data with a browser-based user interface. The nurse followed the patient’s status and the data once a week or more frequently if necessary. In the beginning of the study, the nurse contacted the patient every time the measurement was beyond the target levels or if the patient reported any of the symptoms. Later, the contacts were more dependent on the patient’s measurement history. If the latest measurement markedly differed from previous measurements, the nurse called the patient. The nurse could invite the patient for a check-up visit if still necessary after the phone call. If a patient did not comply with the weekly reporting plan, the nurse contacted the patient and encouraged him or her to continue with the monitoring.

### Outcome Measures

#### Primary Outcome

The primary outcome was the number of HF-related hospital days during the follow-up. The data were obtained from the electronic health record system of HUCH.

#### Secondary Outcomes

Secondary outcomes included clinical outcomes, use of health care resources, and user experience. The following variables were analyzed in order to assess clinical effectiveness: death from any cause, heart transplant operation or listing for transplant operation, left ventricular ejection fraction (LVEF, %) measured by echocardiography, plasma concentration of N-terminal of the prohormone brain natriuretic peptide (NT-proBNP, ng/l), creatinine (μmol/l), sodium (mmol/l), and potassium (mmol/l). For the plasma concentrations of sodium, potassium, and creatinine, there is no unambiguous interpretation of the direction of change, but the value should be within the reference range. The reference ranges used at HUCH are sodium 137-145 mmol/l, potassium 3.3-4.9 mmol/l, creatinine among women 50-90 μmol/l and among men 60-100μmol/l. Sodium, potassium, and creatinine were dichotomized indicating whether the observed value was within the reference range.

Self-care behavior was measured using the European Heart Failure Self-Care Behaviour Scale (EHFSBS). EHFSBS is a 12-item self-administered questionnaire specifically designed and tested for HF patients including statements on self-care behavior essential in the care of HF. The statements are scored from 1-5; the lower the score, the better the performance in self-care. The summary score was analyzed, and medication changes were recorded to examine how the telemonitoring intervention affected activity in medication regimen. The nurse collected information regarding changes in patients’ medication regimen throughout the study. Changes related to medication optimization during the baseline visit were excluded. Changes made to patients’ medication were classified into three categories: increase of medication (a new drug or increase in dosage), decrease of medication (termination of a certain drug or decrease in dosage), and self-imposed medication termination (patient had stopped taking medicine without physician’s confirmation). The medications were classified as diuretics, ACE-I, or beta-blockers.

In terms of the use of health care resources, outpatient visits were analyzed: the number of (1) unplanned visits to the Cardiology Outpatient Clinic (nurse or physician), (2) visits to the emergency department, (3) visits to and time spent with the nurse, (4) visits to and time spent with the physicians, and (5) telephone contacts made by the patient and by the nurse. The baseline visits and the end-point visits were included in the calculations. The data were retrieved from the electronic health records and by asking the patient.

Patients’ acceptance and experience towards home telemonitoring were evaluated using a questionnaire delivered to patients in the telemonitoring group at the end-point visit. The questionnaire included statements about their experiences with the usability of the mobile phone app, as well as their satisfaction with using the app and the benefits of the telemonitoring-assisted care model. In addition, an in-depth interview was conducted with the nurse responsible in order to assess the user experience from a professional perspective.

### Power Calculations

The study was designed to have a power of 90%, an alpha level of .05, and an effect size of 0.5 determined as the expected difference of 3 HF-related hospital days between the study groups (SD 6). A *t* test was used as a calculation framework. With these parameters, we calculated that 44 patients per treatment arm needed to be recruited.

### Statistical Analysis

The intention-to-treat principle was applied in statistical analyses. There was one dropout in the intervention group. The patient withdrew from the study shortly after the beginning, and no end-point measurements were available. The patient was excluded in the end-point analyses. All analyses except zero inflated Poisson (ZIP) were carried out using SPSS version 19. ZIP regression models were conducted using R version 2.15.1.

Outcome variables that express counts (eg, HF-related hospital days, visits to the nurse, visits to the physician, number of phone calls, unplanned visits to the clinic) were presented using the mean and a percentage of zero counts. Poisson regression and ZIP regression models were used in order to analyze the difference between the study groups. The Vuong test [26] was used to assess the superiority between Poisson regression and ZIP for each variable. Finally, ZIP regression was used in the analysis of the following variables: number of HF-related hospital days, number of unplanned visits to the clinic, and telephone contacts initiated by the patient. In all models, the patient’s individual study duration (in days) was set as an offset variable, and the control group was used as a reference group. The incidence rate ratio (IRR) and its 95% confidence interval (Cl) were reported.

Repeated contiguous variables were analyzed within and between the study groups. The paired *t* test or Wilcoxon matched-pair signed-rank test in the case of non-normality was used for the analyses of within-group changes. Non-normality was confirmed by the Kolmogorov-Smirnov test. Analysis of covariance was used to investigate differences between the control and the intervention groups with adjustment for baseline values. The 95% Cl and *P* value for the between-group difference were reported.

##  Results

### Patient Flow

**Figure 3:**
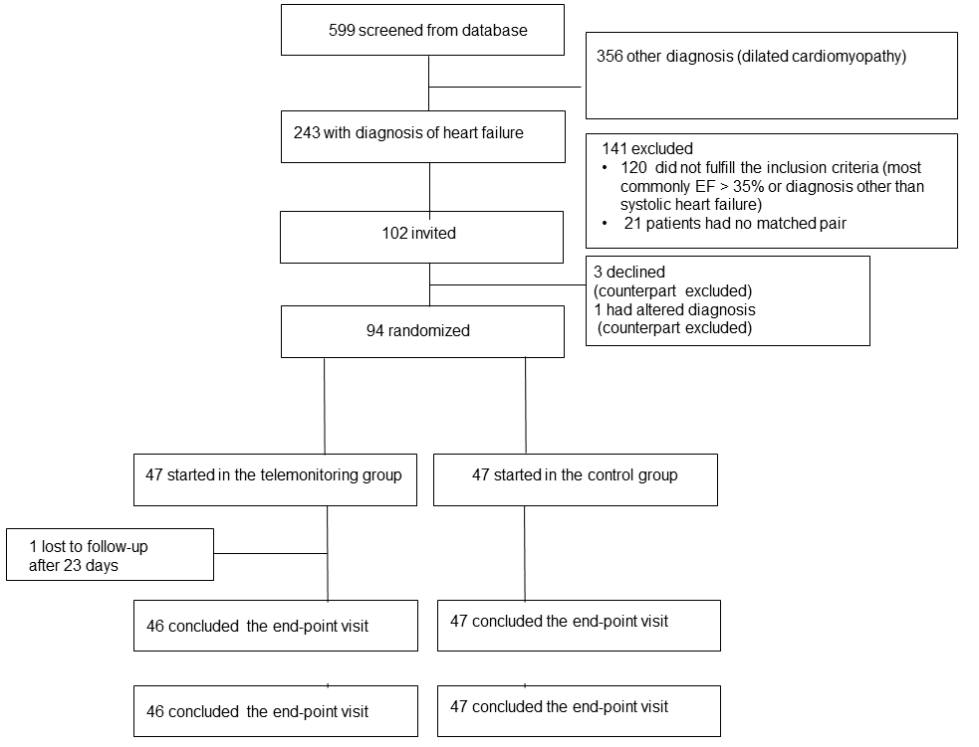
Screening, randomization, and follow-up of patients.

Figure 3 shows the progress of the study. Altogether, 599 patients were screened from the database, of whom 243 were diagnosed with systolic heart failure. Of these, 123 patients fulfilled the inclusion criteria. Eligible patients who were similar in their left ventricular ejection fraction, NYHA classification, age, and gender were matched; 51 matched pairs were identified. The 102 patients were invited for a baseline visit where baseline measurements were taken and information considering the study was given. Of these, 3 patients declined to participate and another patient had a changed diagnosis. Respectively, their matched counterparts were excluded from the study. Finally, 94 patients were randomized. One from each pair was randomly assigned to receive the usual care, and the other was assigned to the telemonitoring group. There was one dropout in the telemonitoring group. The patient withdrew from the study after 23 days. The patient felt that monitoring his condition made him anxious as it reminded him constantly of the disease.

### Baseline Characteristics

Table 1 displays the baseline characteristics of the study subjects in both the control group and the telemonitoring group.

**Table 1:** Baseline characteristics of the patients: mean (standard deviation) or frequency (percentile).

		Control group (n=47)	Telemonitoring group (n=47)
Sex (male), n (%)		39 (83)	39 (83)
Age in years, mean (SD)		57.9 (11.9)	58.3 (11.6)
BMI (kg/m^2^), mean (SD)		27.9 (4.7)	28.4 (6.0)
Systolic blood pressure (mmHg), mean (SD)		116 (16)	112 (13)
Diastolic blood pressure (mmHg), mean (SD)		72 (10)	71 (10)
Heart rate (bpm), mean (SD)		70 (12)	69 (11)
Left ventricular ejection fraction (% units), mean (SD)		28.6 (5.0)	27.3 (4.9)
**NYHA, frequency (%)**			
	Slight limitations in physical activity (Class II)	17 (36)	19 (40)
	Marked limitation in physical activity (Class III)	28 (60)	27 (58)
	Severe limitations in physical activity (Class IV)	2 (4)	1 (2)
**Comorbidities, frequency (%)** ^a^			
	Diabetes	2 (4)	2 (4)
	Hypertension	6 (13)	8 (17)
	Atrial fibrillation	6 (13)	14 (30)
	Asthma/COPD	5 (11)	2 (4)
	Renal insufficiency	4 (9)	0 (2)
	No comorbidities	9 (19)	12 (26)
Smoking (number of non-smokers), n (%) ^a^		42 (89)	35 (76)

^a^Data missing from one patient in the telemonitoring group.

### Primary Outcome

On average there were 1.4 (SD 3.5) HF-related hospital days in the control group and 0.7 (SD 2.4) HF-related hospital days in the telemonitoring group. Of the control patients, 72% (34/47), and of the telemonitoring patients, 83% (38/46) had no hospital days during the 6-month follow-up. The difference between the study groups was not statistically significant (IRR=0.812, 95% Cl 0.525-1.256, *P*=.351).

### Secondary Outcomes

Contrary to expectations, none of the subjects died, underwent a heart transplant operation, or were listed for a transplant operation. In both study groups, two patients had an emergency episode.

Table 2 shows clinical outcomes at baseline and post intervention. There were no statistically significant differences between the study groups in either of the clinical variables. However, in both study groups, there were significant within-group changes: an increase in LVEF (4.2%, *P*=.001 for control group and 5.0%, *P*=.003 for telemonitoring group) and in EHFSBS (-3.8 points, *P*<.001 in the control group and -5.0, *P*<.001 in the telemonitoring group) and a decrease in NT-proBNP levels in the telemonitoring group (-198ng/l, *P*=.01).

**Table 2:** Clinical outcomes at the beginning and the end of the study and the within-group change with the corresponding confidence intervals (*P* value refers to the significance level for the between-group difference).

	Control group (n=46)			Telemonitoring group (n=47)				
	Begin	End	Change (95% Cl)	Begin	End	Change (95% Cl)	Effect size (95% Cl)	*P* value
NT-proBNP (ng/l), median (interquartile range)^a^	1338 (474-2974)	731 (368-2408)	-50 (-831 to 260)	2347 (998-3568)	1014 (609-2631)	-198 (-1921 to 170)	Median difference -148	.256
LVEF (%), mean (SD)	28.6 (5.0)	32.8 (8.2)	4.2 (1.8-6.5)	27.3 (4.9)	32.4 (9.8)	5.0 (1.8-8.1)	β=-.04 (-3.658 to 3.743)	.982
EHFSBS score, mean (SD)	27.9 (6.5)	24.1 (8.3)	-3.8 (-5.4 to -2.1)	27.6 (6.8)	22.6 (6.9)	-5.0 (-7.1 to -3.0)	β=-1.320 (-3.842 to 1.184)	.298
Serum creatinine (μmol/L), median (interquartile range; proportion^b^)^a^	88.0 (78.0-121.0; 0.60)	92.0 (84.0-115.0; 0.57)	2.4 (-2.5 to 6.5)	92.5 (83.8-105.3; 0.67)	95.0 (83.5-112.3; 0.57)	3.5 (-1.0 to 9.0)	Median difference 0.9	.435
Serum potassium (mmol/L), mean (SD; proportion^b^)	4.2 (0.3; 0.98)	4.2 (0.4; 0.98)	-0.06 (-0.18 to 0.05)	4.3 (0.4; 0.94)	4.2 (0.4; 1.0)	-0.1 (-0.3 to 0.01)	β=.008 (-0.141 to 0.156)	.918
Serum sodium (mmol/L), mean (SD; proportion^b^)	140.2 (3.0; 0.96)	140.4 (3.4; 0.85)	0.2 (-0.7 to 1.1)	139.6 (3.0; 0.89)	140.7 (2.5; 0.96)	1.0 (0.2-1.8)	β=-.535 (-1.592 to 0.523)	.318

^a^Non-parametric test.

^b^Proportion of patients whose values were within reference interval.

Changes in medication regimen are presented in Table 3. Significantly more medication changes were done to the patients in the telemonitoring group (*P*=.042 for medication increase and *P*=.026 for medication decrease). All decreases in medication were done to telemonitoring patients, and the decreases were applied to diuretics. The increases in medication in the telemonitoring group involved the following types of medication: five increases in angiotensin converting enzyme inhibitor (ACE-I) therapy, three increases in beta-blockers, and two increases in diuretics. In the control group, the two increases were applied to diuretics.

**Table 3:** Categorized medication adjustments and the number of patients to whom the adjustments were applied.

Adjustments	Control group (n=47)	Telemonitoring group (n=46)	*P* value
Increase in medication, n (%)	2 (4)	8 (17)	.042
Decrease in medication, n (%)	0 (0)	5 (11)	.026
Self-imposed medication termination, n (%)	3 (6)	2 (4)	1.0

Table 4 shows the use of health care resources. The use of the nurse’s resources was significantly greater in the telemonitoring group (mean time at the reception was 48.7 minutes longer, and the number of nurse visits was 1.73 times greater, *P*<.001 and *P*<.001 respectively). There were more telephone contacts between the nurse and the telemonitoring patients (IRR=5.6 for nurse initiated contacts and IRR=1.63 for patient initiated contacts, *P*<.001 and *P*=.049 respectively). Typically the patients called the nurse for information to interpret the monitoring results (eg, the safe range for blood pressure) or because they wanted to make changes to their diuretic medication. The most frequent reason for nurse-induced calls was patients’ non-adherence to self-monitoring: the nurse called patients to remind them to carry out and report the measurements. The number of unplanned visits to the Cardiology Outpatient Clinic was significantly bigger in the telemonitoring group (IRR=3.31, *P*<.001). The control group had on average one unplanned visit to the clinic while telemonitoring patients had 3-4 unplanned visits. The most common reason for unplanned visits was patients’ concern about their worsening condition and the need to discuss it with the nurse. In some cases, patients visited physician reception if they needed immediate help. The reasons for phone calls and unplanned visits were based on nurse’s notes. There was no difference in the use of physician resources: the number of visits and the time used at reception were similar between the study groups.

Depending on the patient’s skills, 10-20 minutes was spent at the baseline visit for educating the patient to use the mobile phone app and blood pressure meter. During the follow-up period, only six telephone calls concerning purely technical problems took place. In three cases, the nurse called the patient during the first days to help them get started with the mobile app. The other three calls were initiated by the patient and were caused due to failed Internet connections. All other contacts took place for medical reasons.

**Table 4:** The use of health care resources per patient during the study.

	Control group (n=47)	Telemonitoring group (n=46)	Effect size (95% CI)	*P* value^a^
Nurse time (minutes), mean (SD)	87 (35)	136 (43)	Mean difference 48.7 (32.5-64.8)	<.001
Number of visits to the nurse, mean (SD)	2.7 (1.0)	4.5 (2.2)	IRR=1.73 (1.38-2.15)	<.001
Physician time (minutes), mean (SD)	69 (23)	76 (34)	Mean difference 6.7 (6.0-18.6)	.340
Number of visits to the physician, mean (SD)	2.0 (0.8)	1.9 (0.9)	IRR=0.95 (0.71-1.28)	.738
Number of telephone contacts initiated by nurse, mean (SD)/[% patients with zero-count]	0.6 (0.9)/(57.4%)	3.0 (2.4)/(15.2%)	IRR=5.6 (3.41-7.63)	<.001
Number of telephone contacts initiated by patient, mean (SD)/[% patients with zero-count]	0.6 (1.5)/(72.3%)	2.3 (2.1)/(30.4%)	IRR=1.63 (0.999-2.66)	<.049
Number of unplanned visits to the Cardiology Outpatient Clinic, mean (SD)/[% patients with zero-count]	1.0 (1.5)/(46.8%)	3.7 (2.6)/(13%)	IRR=3.31 (2.15-5.09)	<.001

^a^Difference between groups.

### Professional Experience

The HF nurse who was involved experienced telemonitoring as a valuable support to the current practice. She reported that the patients of the telemonitoring group took self-measurements more regularly and had internalized the importance of regular self-monitoring. Reception visits were more efficient, since no time was wasted on irrelevant issues. The nurse found that patients had taken their drugs more precisely, although no numerical evidence was collected. The nurse reported that both study groups were more curious about the ongoing study and that patients contacted her more frequently than prior to the study. The benefit that the nurse prioritized was the up-to-date data she received from the patients. The data also provided important support for physicians in their decisions about the patient’s treatment, for example, in terms of adjustments to medication. A potential disadvantage that the nurse brought up was that the measurement data were input by the users; there was a possibility that some users sometimes sent false data by mistake or even intentionally. During the study, however, there were no signs of such problems. Automatic data transfer from monitoring devices would reduce the risk of erroneous data. The nurse responsible for the patients did not see any obstacles in adding telemonitoring as a part of their multidisciplinary care model.

### Patient Experience

Of 46 patients, 44 (96%) responded to the user experience questionnaire. Almost all patients (95%, 42/44) found that making and reporting measurements with the mobile phone app was “very useful” or “quite useful”. The automatic feedback they received after sending the measurements was found to be useful; in fact, 91% (40/44) of patients felt it was “very useful” or “quite useful”. However, 9% (4/44) patients responded that they did not derive any benefit from the feedback. Two thirds (66%, 29/44) responded that the feedback helped them pay more attention to issues essential in the treatment of their disease. In fact, 91% (40/44) of patients responded that the feedback motivated them to take measurements and report them regularly. Just over a quarter of patients (27%, 12/44) reported that the feedback also gave them motivation to change their lifestyle.

Most of the patients accepted the home telemonitoring as part of their care routine. The adherence, calculated as a proportion of weekly submitted self-measurements, was 86% in weight reporting and 89% in blood pressure, heart rate, and symptom reporting. The median number of weight reports was 28 (interquartile range 23-33). The median number of blood pressure and symptom reports was 32 (interquartile range 27-43).

### Post-Hoc Power Calculations

The post-hoc power was calculated using the Poisson model framework. Using the following definitions: exp(beta)=.8, base rate=0.03, total sample=97, mean exposure time=200 days, alpha=.05, R^2^=0 (since the study group was the only predictor), distribution of group allocation= binomial with pi=0.5, the post-hoc power of 0.81 was obtained.

##  Discussion

### Principal Results

This study evaluated whether a multidisciplinary care model would benefit from telemonitoring as an additional element in the care of heart failure patients, primarily in terms of reducing HF-related hospital days. We found that the telemonitoring-assisted care approach led to increased use of health care resources while showing no quantified improvement in the patients’ condition. There was no difference in the number of HF-related hospital days, which was the primary outcome. However, patients and health care providers reacted positively to telemonitoring. Patients’ adherence to the weekly reporting plan was close to 90%, which is high in a population with a severe chronic condition.

The increased use of health care resources was primarily seen in the nurse’s workload. The telemonitoring group had an increased number of visits to the nurse reception, a longer time spent at the reception, and more frequent telephone contacts with the nurse. Similarly, in the studies by Cleland et al [16] and Wade et al [19], home telemonitoring was associated with frequent patient contacts including home and office visits, telephone contacts, and emergency visits. However, in our study there was neither increased need for physician consultation nor increased number of visits to the emergency department. Despite the increased workload, the nurse found the increased number of contacts with patients to be a positive change. In her experience, telemonitoring invoked the patients’ interest in their condition and raised questions that resulted in contacts. The nurse also felt that the control group patients were more active after their enrollment in the study. Patients’ increased curiosity was not reflected in a lower number of HF-related hospitalizations, but we can speculate whether low death rates were associated with this. During the 6-month follow-up, we found no deaths in either of the study groups, which is unexpected since the mortality rate at 30 days after hospital admission is 11% [27], rising to 30% during the first year [3].

When implementing telemonitoring in the care process, the increased workload of care professionals needs to be accounted for. Patients’ increased awareness of their disease is likely to increase contacts. Patients need help in interpreting the monitoring results, and they seek individual advice in order to manage their disease and maintain their enthusiasm. This kind of activity may lead to positive health outcomes during a longer follow-up period than was the case in the present study. It should be carefully considered whether the current resources are able to handle the increased demand, or whether additional personnel should be hired. As Chaundry et al [14] concluded, a telemonitoring strategy would be more effective if embedded in cardiology practices with a greater organizational capacity to implement it. To lessen the increased workload of health care professionals, the potential of active assistance technology is worthy of consideration. Such technologies include highly sophisticated automatic messaging systems providing personalized guidance to patients with minimum involvement of health care personnel. Promising results with active assistance technology have been reported in the care of diabetes patients [28].

In this study, telemonitoring was linked to more individualized care in terms of the pharmacological therapy of HF patients. This shows an important aspect, since optimal pharmacological management reduces morbidity and mortality, but it is complex and objective guidelines are lacking [29]. Significantly more changes in medication regimen were made in the telemonitoring group—medication was increased for 17% of these patients whereas in the control group the corresponding percentage was 4%. In addition, all reductions in medication were done for telemonitoring patients. Reductions were applied to Furosemide, which is a diuretic, indicating successful management of fluid retention. Whether medication changes were the result of self-measurement data that telemonitoring patients provided or through their increased self-care or both cannot be confirmed with these data. The frequency of which the measurements were done may alone not be sufficient to constitute the medication changes, but at least the intervention opened patients’ eyes and raised discussion concerning their medication.

Our negative finding regarding the hospitalization rate is in line with findings in studies [14-20]. However, several studies do show evidence in favor of telemonitoring in the care of HF patients. This brings up the challenge in providing telehealth for the right patients in the right context. In the TIM-HF study, a subgroup analysis revealed that patients with lower depression scores had significantly lower hospitalization and mortality [30]. Comorbidities such as chronic obstructive pulmonary disease, chronic kidney disease, and anemia may have a negative effect by blurring the signal from the monitored variables and thus lowering their predictive value [31]. Furthermore, in their review of telemonitoring for chronic diseases, Pare et al [32] concluded that the beneficial effects of telemonitoring are more consistent in pulmonary and cardiac studies than in diabetes and hypertension. In the tele-HF study [14], the authors concluded that none of the participant characteristics including age, sex, race, LVEF, and NYHA class identified a group in which telemonitoring was more effective. A similar conclusion was drawn in [30] in terms of LVEF, gender, age, or NYHA.

We outline four factors that may be associated with unchanged HF-related hospitalizations rates. First, as multidisciplinary care was part of the care practice at the Cardiology Outpatient Clinic of HUCH, all the patients including the control group received high standard interactive care, and some were already used to home self-monitoring. Second, the study was carried out in the Helsinki area where patients live a short distance from health services. Patients were able to visit the clinic easily without great effort. We found that the most common reason for unplanned visits was that the patient wanted to discuss with the nurse face-to-face the signs of deterioration and worsening condition. Home telemonitoring may be more beneficial when applied in rural areas where patients do not have direct access to health care. Third, our study population was relatively young, and medication for all patients was optimized during the baseline visit. Ejection fraction was on average 28%, which is higher than in the TEN-HMS study [16] of high-risk HF-patients. Telemonitoring may be more efficient among patients with poor prognosis. In the TEN-HMS study, which found home telemonitoring associated with improved survival, patients were older, had severe cardiac dysfunction, were recently hospitalized, and had high mortality rates. Finally, the follow-up time was possibly too short. Improved self-care may be realized as a lower number of hospitalizations after a time interval longer than 6 months.

### Limitations

Post-hoc calculations were conducted based on the Poisson model framework resulting in a power of 0.81, which was less than was determined in initial power calculations. Considering the fact that the 95% confidence interval for the IRR ranged from 0.525 to 1.256, we do not expect that there was a true difference in the number of HF-related hospital days between the study groups, although we did not reach the level of 0.1 for the type II error. However, the predicted difference of 3 days was overestimated since the number of hospital days was 1.4 in the control group. In addition, we note that we conducted multiple hypothesis testing, which increases the probability of falsely rejecting the null hypothesis. However, the statistically significant findings that were seen in the use of health care resources were consistent in several variables supporting each other.

The usage of the nurse’s time was somewhat biased. The time consumed at the baseline visit for the delivery of telemonitoring technology to the patients was counted as time spent by the nurse. Also, when technical problems emerged, patients contacted the nurse. The time used at baseline visit was 10-20 minutes per patient. During the monitoring period, only six contacts were made with the nurse due to technical problems. Therefore, it can be concluded that the time spent on technical issues was marginal and that the increased use of nurse’s resources by telemonitoring patients took place due to medical reasons. Technical issues did not increase the required time to an extent that would lead to significant overestimation. An additional source of bias is the fact that monitoring took place under control of only one research nurse and the professional experience was based only on her interview. Consequently, it is not possible to draw general conclusions on the attitudes of health care professionals on monitoring.

### Conclusions

In the Heart at Home study, we found that home telemonitoring was not efficient to support the multidisciplinary care approach in terms of reducing the number of HF-related hospital days or outpatient visits or improving patients’ clinical condition. The telemonitoring increased significantly the nurse’s workload by increasing the number of reception visits and the number of telephone contacts. The increased workload should be carefully considered when implementing telemonitoring in the care of HF patients. Extra work is required on top of the multidisciplinary care approach. To lessen the increased workload of health care professionals, the potential of active assistance technology is worthy of further consideration to respond to patients’ queries and to keep them motivated.

## Multimedia Appendix 1

CONSORT EHEALTH checklist V1.6.2 [25].

## Abbreviations

ACE.-I: angiotensin converting enzyme inhibitor
EHFSBS: European Heart Failure Self-Care Behaviour Scale
HF: heart failure
HUCH: Helsinki University Central Hospital
IRR: incidence rate ratio
LVEF: left ventricular ejection fraction
NT-proBNP: N-terminal of the prohormone brain natriuretic peptide
NYHA: New York Heart Association
WSD: Whole Systems Demonstrator
