# Supplementary material for: Use of Home Telemonitoring to Support Multidisciplinary Care of Heart Failure Patients in Finland: Randomized Controlled Trial
Source: J Med Internet Res. 2014 Dec 11;16(12):e282. doi: 10.2196/jmir.3651 (PMC4275484; doi:10.2196/jmir.3651)
Supplement: Supplementary file 1 [file jmir_v16i12e282_app1.pdf]

CONSORT-EHEALTH Checklist V1.6.2 Report

(based on CONSORT-EHEALTH V1.6), available at [http://tinyurl.com/consort-ehealth-v1-6].

3651

Date completed

6/26/2014 9:23:12

by

Anna-Leena Orsama

Home telemonitoring to support multidisciplinary care of heart failure patients in Finland: a randomized controlled trial

TITLE

1a-i) Identify the mode of delivery in the title

telemonitoring

1a-ii) Non-web-based components or important co-interventions in title

multidisciplinary care

1a-iii) Primary condition or target group in the title

heart failure patients

ABSTRACT

1b-i) Key features/functionalities/components of the intervention and comparator in the METHODS section of the ABSTRACT

"Patients in the telemonitoring (n=47) group measured their body weight, blood pressure and pulse, and answered symptom-related questions on a weekly basis, reporting their values to the heart failure nurse using a mobile phone application."

1b-ii) Level of human involvement in the METHODS section of the ABSTRACT

"The heart failure nurse followed the status of patients weekly and if necessary contacted the patient."

1b-iii) Open vs. closed, web-based (self-assessment) vs. face-to-face assessments in the METHODS section of the ABSTRACT

"Heart failure patients were eligible whose left ventricular ejection fraction was lower than 35%, NYHA functional class ≥ 2 and who needed regular follow-up. Patients in the telemonitoring (n=47) group measured their body weight, blood pressure and pulse, and answered symptom-related questions on a weekly basis, reporting their values to the heart failure nurse using a mobile phone application. The heart failure nurse followed the status of patients weekly and if necessary contacted the patient. The primary outcome was the number of hospital days at the Cardiology Clinic. Control patients (n=47) received multidisciplinary care that was standard. Patients' clinical status, utilization of health care resources, adherence and user experience from the patients' and the healthcare professionals' perspective were studied."

1b-iv) RESULTS section in abstract must contain use data

"Adherence, calculated as a proportion of weekly submitted self-measurements, was close to 90%. No difference was found in the number of hospital days (Incidence rate ratio (IRR)=0.8, P-value=0.351) which was the primary outcome. The intervention group used significantly more health care resources: they paid an increased number of visits to the nurse (IRR=1.73, P< 0.001), spent more time at the nurse reception (mean difference of 48.7 minutes, P<0.001) and there was a greater number of telephone contacts between the nurse and intervention patients (IRR=3.82, P < 0.001 for nurse-induced contacts and IRR=1.63, P=0.049 for patient-induced contacts)."

1b-v) CONCLUSIONS/DISCUSSION in abstract for negative trials

"Telemonitoring significantly increased the utilization of health care resources without showing benefits in patients' clinical status when compared to the patients who received standard multidisciplinary care."

INTRODUCTION

2a-i) Problem and the type of system/solution

"The objective of this study was to investigate whether the multidisciplinary care of heart failure patients could be promoted with telemonitoring at the Cardiology Clinic of Helsinki University Central Hospital (HUCH). We hypothesized that telemonitoring improves patients' adherence to self-care, something that will be realized as decreased hospitalizations."

2a-ii) Scientific background, rationale: What is known about the (type of) system

"The literature shows conflicting evidence on the effectiveness of telemonitoring dependent on the target population and study environment and the implementation and structure of the intervention itself."

METHODS

3a) CONSORT: Description of trial design (such as parallel, factorial) including allocation ratio

"The objective of this study was to investigate whether the multidisciplinary care of heart failure patients could be promoted with telemonitoring at the Cardiology Clinic of Helsinki University Central Hospital (HUCH). We hypothesized that telemonitoring improves patients' adherence to self-care, something that will be realized as decreased hospitalizations."

3b) CONSORT: Important changes to methods after trial commencement (such as eligibility criteria), with reasons

No changes

3b-i) Bug fixes, Downtimes, Content Changes

No content changes

4a) CONSORT: Eligibility criteria for participants

"The inclusion criteria were: diagnosis of systolic heart failure, aged 18–90 years, NYHA class ≥ 2 (an interview-based classification by the New York Heart Association about the limitations to physical activity), left ventricular ejection fraction ≤ 35% as measured during hospital visits, need for a regular follow-up visit and time from the last visit of less than six months. Patients were not eligible who were known to be about to have a major medical operation, had severe comorbidity such as cancer, had participated in another clinical trial during the last three months or were suspected of poor compliance. "

4a-i) Computer / Internet literacy

"Patients were not eligible who were known to be about to have a major medical operation, had severe comorbidity such as cancer, had participated in another clinical trial during the last three months or were suspected of poor compliance."

4a-ii) Open vs. closed, web-based vs. face-to-face assessments:

"The electronic patient database of HUCH was used for the initial screening of patients with chronic heart failure so as to further assess their eligibility. Eligible patients were informed about the study and they were asked whether they were willing to participate (and their formal consent was obtained) when they came for their normal check-up visit. For willing patients, study-related clinical measurements were taken and the patients completed the study questionnaires. The same procedure was repeated at the end-point visit."

4a-iii) Information giving during recruitment

"Eligible patients were informed about the study and they were asked whether they were willing to participate (and their formal consent was obtained) when they came for their normal check-up visit."

4b) CONSORT: Settings and locations where the data were collected

Study-related clinical measurements were taken and the patients completed the study questionnaires at the baseline visit and end-point visit

4b-i) Report if outcomes were (self-)assessed through online questionnaires

No assessment through online questionnaire.

4b-ii) Report how institutional affiliations are displayed

5) CONSORT: Describe the interventions for each group with sufficient details to allow replication, including how and when they were actually administered

5-i) Mention names, credential, affiliations of the developers, sponsors, and owners

The telemonitoring system was developed by VTT Technical Research Centre of Finland.

5-ii) Describe the history/development process

In the development of the mobile application, particular care was paid to the simplicity of the user interface and its ease of use, since most of the patients were elderly.

5-iii) Revisions and updating

No revisions were made.

5-iv) Quality assurance methods

No

5-v) Ensure replicability by publishing the source code, and/or providing screenshots/screen-capture video, and/or providing flowcharts of the algorithms used

Figure 2 shows screen shots of the reporting process with the software application

5-vi) Digital preservation

5-vii) Access

Mobile phones and applications were provided for patients. The whole process was free.

5-viii) Mode of delivery, features/functionalities/components of the intervention and comparator, and the theoretical framework

"The patients were advised to carry out and report the measurements together with the assessment of symptoms once a week. In the context of each submission of information, the patient received automatic machine-based feedback of whether the reported parameter was within their personal targets set by the cardiac nurse. The overall architecture used in the self-care process and screenshots of the software application are depicted in Figure 1 and Figure 2. The system was developed by VTT Technical Research Centre of Finland."

5-ix) Describe use parameters

""The patients were advised to carry out and report the measurements together with the assessment of symptoms once a week."

5-x) Clarify the level of human involvement

"The measurements were stored on the remote patient monitoring server. The cardiac care team was able to access the data with a browser-based user interface. The responsible nurse followed the patient's status and the data once a week or more frequently if necessary. Based on the self-measurements, the nurse could invite the patient for a check-up visit. If a patient did not take their self-measurements as planned, the nurse contacted the patient and encouraged them to continue with the monitoring."

5-xi) Report any prompts/reminders used

"If a patient did not take their self-measurements as planned, the nurse contacted the patient and encouraged them to continue with the monitoring."

5-xii) Describe any co-interventions (incl. training/support)

All patients received multidisciplinary care.

6a) CONSORT: Completely defined pre-specified primary and secondary outcome measures, including how and when they were assessed

"The primary outcome was the number of HF-related hospital days spent at the HUCH Cardiology Clinic during the study. The data were obtained from the electronic health record system of HUCH.Secondary outcomes include clinical outcomes, utilization of health care resources and user experience. The following variables were analysed in order to assess clinical effectiveness: death from any cause, heart transplant operation or listing for transplant operation, left ventricular ejection fraction (LVEF, %) measured by echocardiography, plasma concentration of brain natriuretic peptide propeptide (P-proBNP, ng/l), creatinine (µmol/l), sodium (mmol/l) and potassium (mmol/l). Concerning the plasma concentrations of sodium, potassium and creatinine, there is no unambiguous interpretation of the direction of change, but the value should be within the reference range. The reference ranges used at HUCH are: sodium 137–145 mmol/l, potassium 3.3–4.9 mmol/l, creatinine among women 50–90 µmol/l, and among men 60–100µmol/l. Sodium, potassium and creatinine were dichotomized indicating whether the observed value is within the reference range. Self-care behaviour was measured using the European Heart Failure Self-Care Behaviour Scale (EHFSBS). EHFSBS is a 12-item self-administered questionnaire specifically designed and tested for heart failure patients including statements on self-care behaviour essential in the care of HF. The statements are scored from one to five. The lower the score, the better the performance in self-care. The summary score is analysed. Data regarding medication regimen and related changes were collected from electronic health records. Changes made to patients' medication were classified into three categories: increase of medication (a new drug or increase in dosage), decrease of medication (termination of a certain drug or decrease in dosage), and self-imposed medication termination (patient had stopped taking medicine without physician's confirmation).

In terms of the utilization of health care resources, outpatient visits were analysed: the number of unplanned visits to the HUCH cardiology clinic (nurse or physician), the number of visits to the emergency clinic, the number of visits to and time spent with the cardiac nurse, the number of visits to and time spent with the cardiac physician, the number of telephone contacts made by the patient and by the nurse. The data were retrieved from the electronic health records and by asking the patient. "

6a-i) Online questionnaires: describe if they were validated for online use and apply CHERRIES items to describe how the questionnaires were designed/deployed

No online questionnaires

6a-ii) Describe whether and how "use" (including intensity of use/dosage) was defined/measured/monitored

The adherence was calculated as a proportion of weekly submitted self-measurements,

6a-iii) Describe whether, how, and when qualitative feedback from participants was obtained

Patients' acceptance and experience were evaluated using a questionnaire delivered to patients in the telemonitoring group at the end-point visit. The questionnaire included statements about their experiences of the usability of the mobile phone application and their satisfaction with and the benefits of the telemonitoring-assisted care model. In addition, an in-depth interview was conducted with the responsible nurse in order to assess the user experience from a professional perspective.

6b) CONSORT: Any changes to trial outcomes after the trial commenced, with reasons

No

7a) CONSORT: How sample size was determined

7a-i) Describe whether and how expected attrition was taken into account when calculating the sample size

Our study was designed to have a power of 90%, an α-level of 0.05 and effect size of 0.5 determined as the expected difference of three hospital days between the study groups (with a standard deviation of six). A t-test was used as a calculation framework. With these parameters, we calculated that 44 patients per treatment arm needed to be recruited.

7b) CONSORT: When applicable, explanation of any interim analyses and stopping guidelines

no stopping rules.

8a) CONSORT: Method used to generate the random allocation sequence

"Matched pair design was used in the randomization. "

8b) CONSORT: Type of randomisation; details of any restriction (such as blocking and block size)

"Matched pair design was used in the randomization. The eligible patients, who were similar as regards their left ventricular ejection fraction, NYHA classification, age and gender, in respective order, were matched in pairs. One was randomized to the control group and the other to the intervention group. "

9) CONSORT: Mechanism used to implement the random allocation sequence (such as sequentially numbered containers), describing any steps taken to conceal the sequence until interventions were assigned

"Patients were matched in pairs. One was randomized to the control group and the other to the intervention group."

10) CONSORT: Who generated the random allocation sequence, who enrolled participants, and who assigned participants to interventions

Study nurse randomly allocated patient pairs in the intervention group and the control group. The randomization was done by flipping a coin prior either of the patients became for baseline visit.

11a) CONSORT: Blinding - If done, who was blinded after assignment to interventions (for example, participants, care providers, those assessing outcomes) and how

11a-i) Specify who was blinded, and who wasn't

Open label. No blinding.

11a-ii) Discuss e.g., whether participants knew which intervention was the "intervention of interest" and which one was the "comparator"

Participant were away of the intervention since they were encouraged for telemonitoring.

11b) CONSORT: If relevant, description of the similarity of interventions

Not relevant.

12a) CONSORT: Statistical methods used to compare groups for primary and secondary outcomes

#Outcome variables that express counts (hospital days, visits to the nurse, visits to the physician, number of phone calls, unplanned visits to the cardiology clinic) were presented using the mean and a percentage of zero counts. Poisson regression and ZIP regression models were used in order to analyse the difference between the study groups. The Vuong [24] test was used to assess the superiority between Poisson regression and ZIP model for each variable. Finally, ZIP regression was used in the analysis of the following variables: number of hospital days, number of unplanned visits to the HUCH Cardio Clinic and telephone contacts initiated by the patient. In all the models, the patient's individual study duration (in days) was set as an offset variable, and the control group was used as a reference group. The incidence rate ratio (IRR) and its 95% confidence interval (95% CI) were reported.

Repeated contiguous variables were analysed within and between the study groups. The paired t-test or Wilcoxon Matched-Pair Signed-Rank test in the case of non-normality were used for the analyses of within-group changes. Non-normality was confirmed by the Kolmogorov-Smirnov test. Analysis of covariance (ANCOVA) was used to investigate differences between the control and the intervention groups with adjustment for baseline values. The 95% CI and P-value for the between-group difference were reported.

For the analysis of binary variables, the logistic regression model was used, where the post intervention level within the target range was the dependent variable. Models were adjusted for patients' baseline levels. The odds ratio (OR) and their 95% CI were reported with the corresponding P-value. Changes in medication regimen were analysed using Fisher's exact test."

12a-i) Imputation techniques to deal with attrition / missing values

"The intention-to-treat (ITT) principle was applied in statistical analyses. There was one drop-out in the intervention group. The patient withdrew from the study shortly after the beginning, and no end-point measurements were available. The patient was excluded in the end-point analyses. "

12b) CONSORT: Methods for additional analyses, such as subgroup analyses and adjusted analyses

All analyses were adjusted was corresponding baseline values.

RESULTS

13a) CONSORT: For each group, the numbers of participants who were randomly assigned, received intended treatment, and were analysed for the primary outcome

Figure 1 shows the progress of the study. Altogether, 890 patients were screened from the database, of whom 123 patients were found to be eligible. Eligible patients who were similar as regards their left ventricular ejection fraction, NYHA classification, age and gender were matched.51 matched pairs were identified. Altogether, 102 patients were invited for a baseline visit in which baseline measures were taken and information was given considering the study. Of these, three declined to participate and one patient had a changed diagnosis. Respectively, their matched counterparts were excluded from the study. "

13b) CONSORT: For each group, losses and exclusions after randomisation, together with reasons

"94 patients were randomized. One from each pair was randomly assigned to receive the usual care and the other was assigned to the telemonitoring group. "

13b-i) Attrition diagram

Only one drop-out during the follow-up.

14a) CONSORT: Dates defining the periods of recruitment and follow-up

The study was divided into two parts. The first 30 intervention patients and 29 control patients started stepwise during November 2010 to February 2011 and were followed for approximately the next six months. After the first 59 patients had finished their follow-up, the second group (17 intervention patients and 18 control patients) started during May–August 2011. This division was made because patients were recruited when they came for their normal control visit and those control visits were spread throughout the year. The nominal follow-up time was six months. The study was completed in February 2012.

14a-i) Indicate if critical "secular events" fell into the study period

No secular events during the trial

14b) CONSORT: Why the trial ended or was stopped (early)

Not relevant

15) CONSORT: A table showing baseline demographic and clinical characteristics for each group

Table 1

15-i) Report demographics associated with digital divide issues

Health literacy was not assessed. However, according the inclusion criteria patients who were suspected of poor compliance were excluded.

16a) CONSORT: For each group, number of participants (denominator) included in each analysis and whether the analysis was by original assigned groups

16-i) Report multiple “denominators” and provide definitions

Numbers are shown in tables.

16-ii) Primary analysis should be intent-to-treat

"The intention-to-treat (ITT) principle was applied in statistical analyses."

17a) CONSORT: For each primary and secondary outcome, results for each group, and the estimated effect size and its precision (such as 95% confidence interval)

effect size and 96% CI's are reported in tables

17a-i) Presentation of process outcomes such as metrics of use and intensity of use

Adherence is reported

17b) CONSORT: For binary outcomes, presentation of both absolute and relative effect sizes is recommended

Only relative effect sizes are reported because lack of space.

18) CONSORT: Results of any other analyses performed, including subgroup analyses and adjusted analyses, distinguishing pre-specified from exploratory

No subgroup analyses.

18-i) Subgroup analysis of comparing only users

No subgroup analyses.

19) CONSORT: All important harms or unintended effects in each group

No unintended effects

19-i) Include privacy breaches, technical problems

No privacy problems reported by patients or by health care staff.

19-ii) Include qualitative feedback from participants or observations from staff/researchers

"Professional experience

The responsible HF nurse experienced telemonitoring as a valuable support to the current practice. She reported that the patients of the telemonitoring group took self-measurements more regularly and had internalized the importance of regular self-monitoring. Reception visits were more efficient, since no time was wasted on irrelevant issues. The nurse experienced that patients had taken their drugs more precisely, although no numerical evidence was collected. The nurse found that both study groups were more curious about the ongoing study and that patients contacted her more frequently than prior to the study. The benefit that the nurse prioritized was the up-to-date data she received from the patients. The data also provided important support for physicians in their decisions about the patient’s treatment, for example in terms of adjustments to medication. A potential disadvantage that the nurse brought up was that the measurement data is input by the users and there is a possibility that some users sometimes send false data by mistake or even intentionally. During the study there were no signs of such problems. Automatic data transfer from monitoring devices would reduce the risk of erroneous data. The responsible nurse did not see any obstacles to adding telemonitoring as a part of their multidisciplinary care model.

Patient experience

Forty-four patients responded to the user experience questionnaire. Almost all the patients (95%) found that making and reporting measurements with the mobile phone application was ‘useful’ or ‘quite useful’. The automatic feedback they received after sending the measurements was found to be useful – 91% of patients felt it ‘very useful’ or ‘quite useful’. Four patients responded that they did not derive any benefit from the feedback. Two thirds (66%) responded that the feedback helped them to pay attention to issues essential in the treatment of their disease. Forty patients (91%) responded that the feedback motivated them to take measurements and report them regularly. For twelve (27%) patients, the feedback also provided motivation to change their lifestyle and make it healthier."

DISCUSSION

20) CONSORT: Trial limitations, addressing sources of potential bias, imprecision, multiplicity of analyses

20-i) Typical limitations in ehealth trials

"Post hoc calculations were conducted based on the Poisson model framework resulting in a power of 0.81, which was less than was determined in initial power calculations. Considering the fact that the 95% confidence interval for the IRR ranged from 0.525 to 1.256, we do not expect that there was a true difference in the number of hospital days between the study groups, although we did not reach the level of 0.1 for the type II error.

The usage of the nurse's time was somewhat biased. The time consumed at the baseline visit for the delivery of telemonitoring technology to the patients was counted as time spent by the nurse. Also, when technical problems emerged, patients contacted the nurse. Once the technology becomes more mature and telemonitoring is used routinely, the time for implementing the technology will be relatively shorter and technical problems will be handled by a technical team rather than by health care staff. Therefore, the required nurse time for the telemonitoring patients was overestimated in the study. Additionally, the study involved only one research nurse and the professional experience was based only on her interview. This certainly limits the generalization of professionals' perspectives.

We note that we conducted multiple hypothesis testing which increases the probability of falsely rejecting the null hypothesis. However, the statistically significant findings that were made in the use of health care resources were consistent in several variables supporting each other. Also, the number of the changes in medication that were found to be significantly larger in the telemonitoring group was also supported by the nurse's experience. These indicating that the findings did not occur by chance."

21) CONSORT: Generalisability (external validity, applicability) of the trial findings

21-i) Generalizability to other populations

21-ii) Discuss if there were elements in the RCT that would be different in a routine application setting

The increasing workload is a point to take under careful consideration when implementing telemonitoring in the care of HF patients. Extra work is required on top of the multidisciplinary care approach.

22) CONSORT: Interpretation consistent with results, balancing benefits and harms, and considering other relevant evidence

22-i) Restate study questions and summarize the answers suggested by the data, starting with primary outcomes and process outcomes (use)

"This study evaluated whether a multidisciplinary care model would benefit from telemonitoring as an additional element in the care of heart failure patients. We found that the telemonitoring-assisted care approach significantly increased the use of health care resources, but showed no improvement in the patients' conditions. There was no difference in the number of hospital days, which was the primary outcome. However, patients and health care providers reacted positively to telemonitoring. Patients' adherence to the weekly reporting plan was close to 90%, which is high in this elderly study population with a severe chronic condition. "

22-ii) Highlight unanswered new questions, suggest future research

"Whether medication changes were the result of self-measurement data that telemonitoring patients provided or through their increased self-care or both cannot be confirmed with these data. However, the nurse experienced that the data were helpful and supported her work."

Other information

23) CONSORT: Registration number and name of trial registry

clinicaltrials.gov

24) CONSORT: Where the full trial protocol can be accessed, if available

Protocol is not available.

25) CONSORT: Sources of funding and other support (such as supply of drugs), role of funders

We wish to thank the personnel of the Cardiology Clinic of HUCH, and in particular research nurse Leila Partanen, for carrying out the clinical part of the study. The Finnish Funding Agency for Technology and Innovation and VTT Technical Research Centre of Finland are acknowledged for funding the study.

X26-i) Comment on ethics committee approval

"The study protocol was approved by the Ethics Committee of the Hospital District of Helsinki and Uusimaa."

x26-ii) Outline informed consent procedures

"All the patients provided a written informed consent before they were randomized."
X26-iii) Safety and security procedures

X27-i) State the relation of the study team towards the system being evaluated
VTT Technical Reserach Centre of Finland developed the telemonitoring system.
